# Navigating the bench to bedside maze: Lessons from CTSA hubs on streamlining research support in academic medical centers

**DOI:** 10.1017/cts.2025.10118

**Published:** 2025-08-07

**Authors:** Leah Pope, Bernard Chang, Michelle McClave, Sheila Marie O’Byrne, Kawthar Muhammad, Zainab Abedin, Mary Purcell, Anjana Nair, Kayla Zalcgendler, Muredach P. Reilly

**Affiliations:** 1 Department of Psychiatry, Columbia University, Vagelos College of Physicians and Surgeons, New York State Psychiatric Institute, Irving Institute for Clinical and Translational Research, New York, NY, USA; 2 Department of Emergency Medicine, Irving Institute for Clinical and Translational Research, Columbia University Irving Medical Center, New York, NY, USA; 3 Irving Institute for Clinical and Translational Research, Columbia University Irving Medical Center, New York, NY, USA; 4 Department of Cardiology, Irving Institute for Clinical and Translational Research, Columbia University Irving Medical Center, New York, NY, USA

**Keywords:** CTSA, research navigation, resource management, training, web site development

## Abstract

**Background:**

Academic Medical Centers (AMCs) with Clinical and Translational Science Awards (CTSAs) offer a range of resources to support clinical and translational research and science. However, research professionals often face challenges in navigating these resources effectively.

**Objective:**

Our study sought to examine research navigation services across CTSA hubs to identify successful strategies, common challenges, and best practices for supporting research teams.

**Methods:**

We conducted interviews with representatives from ten CTSA hubs and performed a landscape analysis to explore the types of research navigation services available, the methods of advertising and orienting faculty and staff, and the challenges faced in launching and maintaining these services.

**Results:**

Our analysis identified three primary types of research navigation services offered at CTSA hubs: online resource libraries, personalized research navigation with dedicated staff, and interdisciplinary research “studios” for protocol development. Despite these offerings, challenges such as low awareness, difficulties coordinating across siloed university systems, and limited metrics for evaluating navigation services persist.

**Conclusion:**

Effective research navigation requires a combination of web-based applications and in-person support, backed by institutional commitment to foster engagement and streamline access. Key strategies for successful navigation services include proactive advertising, integration with orientation programs, and cross-departmental collaboration. Our findings offer actionable recommendations for enhancing research navigation at AMCs, ultimately aiming to increase research productivity and collaboration.

## Introduction

Academic medical centers (AMCs) provide a wealth of research support services and resources to teams across the professional spectrum including clinician-scientists, research scientists, research coordinators, and research administrators. However, navigating and understanding the full range of available support including consultations, funding, and expertise, is a significant challenge for research professionals. Academic medical centers with a Clinical and Translational Science Award (CTSA), funded by the National Center for Advancing Translational Science (NCATS), have an additional mandate and opportunity to facilitate research as part of a national network of medical institutions that “speeds the translation of research discoveries into improved care” [1]. CTSA Program Goals #1 and #4 [1] are in direct alignment with developing and supporting efficient and effective research:

1. Advance clinical and translational science: develop, demonstrate and disseminate scientific and operational innovations that improve the efficiency and effectiveness of clinical translation from identification to first-in-human studies to medical practice implementation to community health dissemination.
2. Create and implement scientific and operational innovations that increase the quality, safety, efficiency, effectiveness and informativeness of clinical research.

The Irving Institute for Clinical and Translational Research is home to Columbia University’s CTSA grant. The Irving Institute is within the Vagelos College of Physicians and Surgeons at Columbia University Irving Medical Center (CUIMC). At our CTSA Program hub there is increasing recognition of the need to systematically equip research professionals with the tools and resources they need to navigate the research landscape and accelerate discovery.

A literature review of the approaches taken by other CTSA hubs to support Research Navigation reveals a diverse range of strategies and programs, with some tailored to specific research focuses. In some institutions, artificial intelligence is being used to streamline research processes and overcome challenges such as data management and analysis [2]. Other institutions have developed a series of tools and resources such as the interactive roadmap myRESEARCHpath [3] and CTSA Search Solutions [2,4]. Web-based applications and tools across AMCs allow for greater collaboration and stakeholder engagement to enhance cross-team collaborations [5,6]. Some institutions have implemented approaches to Research Navigation with more personalized interactions such as research consultation hotlines [7] or structured multidisciplinary initiatives and internal support programs that bring together various experts to provide comprehensive research support [8–10]. The impact and importance of education and workforce development and competency-based career navigation services has also been noted as a key factor for success [11–13].

Despite the availability of robust navigation systems and evidence of researchers’ success in using them, research support services are underused at many AMCs and CTSA Program hubs [14]. Our own research into the utilization of research resources at CTSA hubs found that awareness and ease of access are critical factors [15]. Challenges underscore the importance of continuous improvement in research navigation services to address evolving needs and barriers. These services and systems can be critical tools to support clinical and translational research programs but depend on ongoing improvements in communication, collaboration, and education.

To guide the development of a Research Navigation program at Columbia University’s Irving Institute of Clinical and Translational Research CTSA, we conducted a landscape analysis of Research Navigation services across CTSA hubs in the United States and interviewed representatives from 10 hubs. In this publication, we share insights gained from these interviews, focusing on the types of Research Navigation services offered to research professionals, orientation to these services, and the challenges faced by CTSAs in launching and maintaining these initiatives. We also explore the specific difficulties these hubs encountered in raising awareness within their research communities and conclude with lessons learned and best practices for enhancing research navigation services.

## Methods

Beginning in September 2023, we recruited CTSA administrative leadership for interviews about their Research Navigation services. An initial list of 14 CTSAs to contact was assembled based on the results of a landscape analysis of 66 CTSA Program Hub websites conducted between December 2021 and February 2022 [15]. That landscape analysis revealed that 45 CTSAs (68%) had some form of research navigation service, although there was considerable variability in the types of services offered. We selected 14 hubs through purposeful sampling based on features of their research navigation services that were in alignment with our vision for Research Navigation at our CTSA Program Hub – balancing features that would be practical for implementation in the near term with those that were more aspirational and appropriate for longer term planning. The research team emailed primary contacts at the 14 CTSA hubs to request interviews. Of those emailed, 10 agreed to participate in an interview. Four Irving Institute staff conducted interviews with CTSA administrators between September and November 2023, with at least two staff participating in each interview, one primary interviewer and one primary note taker. Administrators – people in positions of oversight/leadership (e.g., Administrative Directors, Program Managers, Evaluation Directors) – were selected rather than staff providing direct navigation services because we wanted to ensure broad understanding of why and how the services were developed at each CTSA rather than only understanding the types of resources or services that investigators seek. Interviews were designed to allow each CTSA to provide an overview of their research navigation services and the infrastructure that supports them, discuss how services are advertised, how staff are oriented to them, comment on any evaluation of the service, and reflect on challenges, successes, and lessons learned in launching and implementing their service. Interviews were conducted via Zoom, lasted approximately one hour, and were audio-recorded. The protocol for this study was submitted and approved by the Columbia University Institutional Review Board (AAAU5309). Consent, both written and verbal, was received from the individuals interviewed prior to the recording.

Audio recordings were transcribed in preparation for analysis. One co-author, a PhD-level qualitative researcher, read all transcripts and developed a structured template for coding and analysis based on the primary initial categories of the interview. This primarily deductive approach draws on Crabtree and Miller’s (2022) template approach [16] and guidance from Bingham (2023) [17] to organize the data according to study goals and research questions. Data that was originally identified as an “implementation challenge” was then further subjected to open coding to identify emerging topics. Findings were presented back to the research team for further discussion and refinement and triangulated with the data obtained from the landscape analysis to increase the trustworthiness of the findings.

## Results

CTSAs that participated in this study represent institutions of varying sizes and from diverse geographic locations. Table 1 characterizes participating CTSAs according to their geographic region, size, and services offered. CTSA size is based on the 5-year average of the most current National Institute of Health (NIH) Direct Costs of the CTSA applicant institution plus any partners. The NCATS categorized budget tiers for hubs applying for CTSA funding starting in January 2025 as A (> $385,000,000), C ($250,000,000–$384,999,999), T ($175,000,000–$249,999,999), and G (<$174,999,999). Below we report further on the types of research navigation services these CTSAs provide, how services are advertised, and how faculty and staff are oriented to them. We then describe a series of implementation challenges, lessons learned, and suggestions for improvement.

**Table 1.** Overview of participating CTSAs and research navigation services offered

CTSA	Location	5-year NIH Direct Cost Funding Average (FY19-FY23)	Hub Tier	Service Offered			
				Public Resource Library	Internal Resource Library/Portal	Research Navigators	Research Studio/ Development Team
CTSA 1	South	$74,762,644	G	x	x	x	
CTSA 2	South	$436,034,438	A	x	x	x	
CTSA 3	Northeast	$281,010,010	C	x		x	
CTSA 4	Northeast	$350,505,182	C			x	x
CTSA 5	West	$222,157,183	T			x	x
CTSA 6	Northeast	$125,798,634	G			x	
CTSA 7	Northeast	$273,375,458	C		x	x	x
CTSA 8	Northeast	$221,981,581	T	x			
CTSA 9	South	$405,141,035	A		x	x	
CTSA 10	South	$258,531,182	C		x	x	x

### Types of services

CTSAs reported three primary types of services offered through their programs (see Table 1). Services are generally funded through a combination of funds from the CTSA and internal institutional support.

First, the majority of the participating CTSAs (*n* = 7) have developed online resource libraries or self-service portals through which researchers can navigate the research environment at their institutions. These libraries are designed as central hubs for integrating knowledge and resources and may be public-facing or internal systems that require verification of a user’s affiliation to the institution through logging in with a username and password. For example, such libraries might have links to relevant research offices, including commonly used forms or policies, or guide investigators through initial protocol development. At two of the CTSAs, these resource libraries also contain features that allow investigators to create a personalized roadmap based on their specific study needs.

Second, almost all of the CTSAs we interviewed (*n* = 9) have developed a more personalized Research Navigation service that includes dedicated staff who can answer investigator questions and guide them to appropriate resources. Some institutions have full-time navigators assigned to staff this service and others have multiple people who each dedicate a portion of their time to serving as navigators. CTSAs employ a variety of ticketing systems to accept questions/requests and triage them appropriately, with some institutions relying on homegrown systems and others using more widely available software (e.g., REDCap, Service Now, Teamwork).

Finally, almost half of the participating CTSAs (*n* = 4) have developed more intensive research “studios” to support investigators in developing their research protocols. This includes opportunities such as: (1) mock study sections for investigators developing protocols; (2) interdisciplinary navigation meetings to advise investigators on the science of their protocol or on operational aspects and logistics prior to IRB submission; or (3) study initiation meetings after IRB approval is obtained and prior to participant enrollment. At one institution, for example, the CTSA studio service coordinates and hosts a series of interdisciplinary navigation meetings for investigators once they have developed their protocol. This provides a forum for feedback from relevant project stakeholders (e.g., IRB staff, nursing staff, faculty with relevant expertise). This CTSA hub described that it usually takes approximately four meetings for the investigator to be ready for IRB submission. The success of the process has led the CTSA to require it for all clinical scholars submitting their first IRB protocol at the institution.

### Advertising and orientation

CTSAs described relying on a range of strategies to advertise their Research Navigation program and orient faculty and staff to the available research support services. The most common advertising strategy was the development of presentations that the Research Navigation team provides in a variety of settings such as orientations, faculty meetings, departmental meetings, and research coordinator meetings. New faculty orientation was noted by several institutions as a time of high opportunity to build awareness of available support services. A range of outreach and orientation tactics were described including partnering with offices across the research infrastructure beyond the CTSA to host orientation sessions on a monthly, biannual, or annual basis. Some institutions offer more intensive services for new faculty. For example, two institutions described mandatory onboarding sessions for new faculty that are designed to be more personalized to fit the specific research needs of each investigator. Another institution hosts a faculty boot camp for early career and new faculty that meets monthly for one year. Finally, as specific research navigation tools have been developed (e.g., personalized research roadmap online), some CTSAs offer weekly orientations so that all faculty and staff can be trained in how to use them. One CTSA described setting up symposia, as well as “cafes” targeted toward research staff (e.g., study coordinators, research nurses) in which tables are set up for each consultation service and research staff can learn about services and network with other people in similar roles.

CTSAs also use more passive strategies, such as announcing their services through their regular email newsletters and social media or by embedding prominent links on their website or email signatures.

### Challenges in launching research navigation services

Five primary challenges in the implementation of Research Navigation services emerged from interviews: (1) limited awareness about the navigation service and CTSA services in general (among CTSA end users/researchers/trainees); (2) coordination across large, siloed AMC systems; (3) limited faculty and staff engagement with the available online tools; (4) sufficient staffing and knowledge required for the service; and (5) gathering meaningful feedback on navigation services.

First, several of the CTSAs described an ongoing challenge of making faculty and staff aware of their research navigation services or CTSA-supported services in general. As one interview participant described, “one of the problems that we continue to struggle with is telling people we exist… it’s kind of a branding issue and so just awareness, constantly trying to bring awareness to what the CTSA does and how we can help is something we continue to try to achieve.” This was echoed by other interviewees who commented on researchers’ lack of awareness about services or about the fact that researchers may not know that a specific service they have used is nested within a larger CTSA infrastructure. Interviewees described how lack of awareness has led to inefficiencies and duplication of effort in the past and how there is increased focus on “transparency and connectivity” to ensure that the CTSA is able to roll out consistent information across a large institution.

The problem of awareness highlights a second challenge that interview participants raised regarding launching research navigation services: introducing a new service within a large and often siloed AMC. The research enterprise inherently involves coordination across multiple university offices (e.g., grants, contracts, human subjects research protection, purchasing, human resources, etc.) and many of the CTSAs interviewed sit within universities that have a separate office for research infrastructure. As a result, information exists in multiple places and databases and getting a variety of parties on board to create a centralized and streamlined research navigation service that could serve a broad set of constituents to sustain commitment and coordination. One interview participant reflected the following about their experience:

As you can imagine in an institution that’s large but siloed, it was really challenging to get folks on board to coordinate all the resources… getting those offices like grants and contracts, the business office, the office of protection of human subjects and IRB compliance, all of them working together was immensely challenging.

Interview participants also noted the challenge of getting researchers to use the web-based research navigation applications and tools that their CTSAs have created (e.g., online resource libraries or self-service portals). Some offered that this may be related to user interface design issues. For example, one CTSA noted that their website is “not as intuitive as it could be.” A second CTSA described how they were “trying to take a step back and look at how investigators get access to our services to make it easier on them… [and reduce] the length of time it takes you to see the programs you need.” A third CTSA also described the challenge of making decisions about how to “bucket” services, “the other thing we have problems with is figuring out how to put things into buckets. We have so many resources that could be in all buckets. We want them to think about recruitment at every step of the process. So, what do you do with it? Right, where do you put it?” Beyond technical and design issues, however, other CTSAs noted that some investigators and staff may continue to prefer picking up the phone to get answers to their questions. Therefore, having an online tool for researchers may not decrease the need for staff that can work with investigators on an individual basis.

Indeed, a fourth challenge is the fact that such a service is a labor-intensive endeavor. As one interviewee reflected, “Research Navigation is a ‘people heavy’ role.” Websites and web-based applications require constant updates to ensure that information and guidance is current. More personalized services require staff time and networking to ensure that the individuals who serve as research navigators “independently have connections into the right offices and into the right places.” One CTSA remarked, “the strong suit is our personal touch… [but] our strength is also a little bit of our weakness.” Another CTSA described how the value of a human research navigator could not be replaced by a web-based application or tool:

Those networking relationships that people have are really important. And as much as I would like to say, those could be replaced by an informatics tool of some sort or a comprehensive web page that gets people to the right places, I’ve yet to see that done successfully. So yes, I think it is a very people heavy type of role that is needed to do this.

Recognizing the human effort required to make these navigation services successful has led several of the CTSAs to consider ways to increase efficiency. For example, two CTSAs described new processes in place to ensure their online research services remain up to date. At one CTSA, they have created a semi-automatic process to send website information to relevant offices for review and update it on a recurring basis (eliminating the need for research navigators to email individual offices regularly). At a second CTSA, they have created a Research Navigation task force composed of a point person from each of the offices involved in the research process to ensure that information is current.

A final challenge is how to meaningfully evaluate research navigation services. Most of the CTSAs we interviewed said they had the capacity to track website usage and to track the number and type of requests for support they received, as well as who is requesting these services (e.g., principal investigators, research coordinators). In some cases, the CTSAs are using this data to understand what services are being used and where additional support might be needed. For example, one CTSA described how they might decide to do a larger training on a certain topic (e.g., obtaining informed consent) if they received multiple requests on the same topic. Beyond tracking requests, some of the CTSAs have incorporated satisfaction surveys that are sent to individuals who submit a request for assistance. However, these surveys may be sporadic rather than sent automatically and the CTSAs that mentioned administering these surveys mostly concluded they were of limited value, noting that “response rates can be terrible” or that they “very rarely get filled out… [and are] not a very good way to collect information.” Several interview participants also acknowledged wanting to do more in terms of evaluation – whether identifying new feedback mechanisms or thinking critically about the appropriate metrics for evaluating research navigation services. One interviewee acknowledged the challenge of thinking through an appropriate evaluation plan:

What are the metrics for navigation? Because really when it comes down to it, what we’re doing is connecting people…and how do you measure that? Do you measure whether or not they use the service? Do you measure their satisfaction with the service? That feels wrong because you know, we don’t really control the service itself. Do we measure the satisfaction of the people having been connected? Was it timely? Was it courteous? You know, is it more like a customer service type of thing that we’re measuring.

A few additional CTSAs mentioned they were in the process of reconsidering how they evaluate their research navigation services given the limited meaningful feedback they have been able to collect to date.

## Discussion

Our interviews with CTSA hubs have provided valuable insights that will guide us in developing a Research Navigation program at CUIMC. Our study’s sample size was small (10 out of 66 CTSA hubs) and did not include representation from CTSA hubs located in the Midwest or Southeast, thus potentially biasing our findings. However, the consistency of themes across interviews and regardless of geography give us confidence in using our study results to inform efforts at CUIMC.

Many barriers that research professionals face at AMCs extend beyond the scope of the CTSA hub’s influence. Often, CTSA hubs lack the authority and resources – such as staff, funding, time, expertise, and technology – to implement the changes needed to overcome these barriers. Addressing these foundational challenges requires a concerted effort to identify and resolve inefficiencies within the institution’s research processes and structures.

Institutional offices and services, such as the Institutional Review Board, purchasing, and human resources, are often vast, complex, and siloed, making them difficult to navigate. Institutional initiatives are essential to assess these services, identify needs, and enact changes that foster collaboration and dismantle perceived or real barriers. Additionally, dedicated institutional funding is crucial to support the growth and sustainability of a comprehensive Research Navigation service.

We have identified a variety of approaches to delivering research services, each suited to different needs within the research community. A mixed-methods approach is ideal, incorporating both web-based applications and personalized support. Web-based applications – such as websites, online libraries, and research roadmaps – play an essential role in guiding researchers through stages from protocol development and training to grant submission. However, many hubs report that a personal touch remains highly valued. In-person office hours, consultation services, studio sessions, and actively monitored research navigation hotlines or phone support provide essential, tailored assistance. These services require dedicated resources, from keeping content current to coordinating meetings and responding to inquiries.

Creating awareness of these services is critical to meeting research professionals’ needs at all career stages. Studies show that available resources are often underutilized due to lack of awareness or hesitation to engage with new online tools [14]. To address this, a strategic marketing and outreach plan is essential, with ongoing efforts to promote services across the institution. Several hubs incorporate research navigation presentations into new faculty and staff orientations, regular departmental meetings, and targeted events. A range of strategies, from email announcements to educational symposia, can encourage institutional engagement with research navigation services.

Equally important is the development of ongoing evaluation methods to assess the effectiveness of these services and adapt them over time. Moving forward, conducting needs assessments to identify specific and targeted areas where researchers would benefit from enhanced navigation support is essential. We plan to evaluate the effectiveness of our web-based applications and tools to determine how we might improve them and whether increased in-person interaction would improve user experience. We are also scheduling and hosting focus groups to engage with various cohorts of researchers, faculty, and staff at different career stages to learn about their needs and gather feedback on our research navigation system. Additionally, we intend to use listening sessions and periodic surveys to gather feedback. Our interviews underscore that maintaining an effective research navigation service is a continuous, iterative process requiring regular updates to stay relevant and responsive to evolving research needs.

## Conclusion

Building out Research Navigation services within CTSA hubs and AMCs is one strategy for helping research professionals navigate the breadth of resources that they can leverage to increase research productivity and collaboration. Our research makes clear that successful and effective Research Navigation services require resources and investment in web-based applications and tools, as well as staff who are knowledgeable about the conduct of clinical and translational research and have strong networks of connections across the institution. Broad institutional involvement is also pivotal to identifying the barriers impacting research efficiency and ensuring commitment to continuous quality improvement and iteration of solutions. Future work should continue to track the range of operational innovations that CTSAs are developing to address barriers in the efficiency, quality, and effectiveness of clinical and translational research and science, to ultimately speed the translation of research discoveries to improve health.

## References

[1] National Center for Advancing Translational Sciences. Clinician and Translational Science Awards (CTSA) Program. National Center for Advancing Translational Sciences. (https://ncats.nih.gov/research/research-activities/ctsa) Accessed June 6, 2025.

[2] Bernstam EV , Smith JW , Johnson KB , et al. Artificial intelligence in clinical and translational science: successes, challenges and opportunities. Clin Transl Sci. 2022;15:309–321. doi: 10.1111/cts.13164. 34706145 PMC8841416

[3] Wylie J , Tcheng JE , Gordon AM , et al. 518 myRESEARCHpath: an interactive roadmap for navigating research process, resources, and policies at Duke university. J Clin Transl Sci. 2022;6:107–107.

[4] Tafuto B , Vyas R , Pruis T. CTSA search solutions: a novel approach to searching CTSA hub website content. J Clin Transl Sci. 2022;6:e132. 36756074 10.1017/cts.2022.485 PMC9879907

[5] Kwan BM , Rockwood A , Borrayo E , et al. A stakeholder engagement method navigator webtool for clinical and translational science. J Clin Transl Sci. 2021;5:e180. 34849255 10.1017/cts.2021.850 PMC8596067

[6] Swartz K , Brisson C , Zamboni S , et al. 524 navigator: providing a foundation for cross team collaboration and custom research service through the CTSA hub. J Clin Transl Sci. 2024;8:155–155.

[7] Brouwer RN , Voss M , Kaizer AM , et al. Supporting researchers through full-service hotline and consultation services: success in simplicity, customization, and staffing. J Clin Transl Sci. 2020;4:3–7. 32257404 10.1017/cts.2019.428 PMC7103471

[8] Brassil D , Benvenuti M , Scott R , et al. The Rockefeller university navigation program: a structured multidisciplinary protocol development and educational program to advance translational research. Clin Transl Sci. 2014;7:12–19. 24405608 10.1111/cts.12134 PMC4074027

[9] Byrne DW , Vanderpool RC , Pulley JM , et al. Clinical and translational research studios: a multidisciplinary internal support program. Acad Med. 2012;87:1052–1059. 22722360 10.1097/ACM.0b013e31825d29d4 PMC3406254

[10] Snyder DC , Greenberg SB , Gagliano RA Jr , et al. Retooling institutional support infrastructure for clinical research. Contemp Clin Trials. 2016;48:139–145. 27125563 10.1016/j.cct.2016.04.010 PMC4889441

[11] Loucks TL , Meagher EA , Fyffe DC , et al. Clinical and translational research workforce education survey identifies needs of faculty and staff. J Clin Transl Sci. 2022;6:e8. 35211334 10.1017/cts.2021.875 PMC8826003

[12] Hamdawi M , Keyes A. 520 Johns Hopkins institute for clinical and translational research (ICTR) - research personnel onboarding program. J Clin Transl Sci. 2024;8:154–155.

[13] Choi I , Johnson S , Benson K , et al. Conceptualization, development, and early dissemination of eMPACTTM: a competency-based career navigation system for translational research professionals. J Clin Transl Sci. 2024;8:e2. 38384909 10.1017/cts.2023.693 PMC10879852

[14] Elworth JT , Li L , Barbour R , et al. Exploratory study of the underutilization of CTSA module services. J Clin Transl Sci. 2022;6:e114. 36285017 10.1017/cts.2022.440 PMC9549576

[15] McClave-Liu M , Lee L , Chen J , et al. 404 if you build it, will they come? Navigating research resources at CTSA hubs. J Clin Transl Sci. 2023;7:120–120.

[16] Miller CA. Doing Qualitative Research. Sage Publications, 2022.

[17] Bingham AJ. From data management to actionable findings: a five-phase process of qualitative data analysis. Qual Inq. 2023;22:16094069231183620. doi: 10.1177/16094069231183620.

